# A systematic review comparing the results of early vs delayed ligament surgeries in single anterior cruciate ligament and multiligament knee injuries

**DOI:** 10.1186/s43019-020-00086-9

**Published:** 2021-01-07

**Authors:** Seong Hwan Kim, Sang-Jin Han, Yong-Beom Park, Dong-Hyun Kim, Han-Jun Lee, Nicolas Pujol

**Affiliations:** 1 Department of Orthopedic Surgery, Hyundae General Hospital, Chung-Ang University College of Medicine, Jinjeop-eup, Namyangju-si, Gyunggi-do Republic of Korea; 2 Department of Orthopedic Surgery, Chung-Ang University Hospital, Chung-Ang University College of Medicine, 102 Heukseok-ro, Dongjak-gu, Seoul, 06973 Republic of Korea; 3 Department of Orthopedic Surgery, Centre Hospitalier de Versailles, Le Chesnay, France

**Keywords:** Early surgery, Delayed surgery, Multiligament knee injury, Anterior cruciate ligament injury, Meta-analysis

## Abstract

**Purpose:**

The purpose of this study was to compare clinical outcomes and incidence of concomitant injuries in patients undergoing early vs delayed surgical treatment of single anterior cruciate ligament (ACL) injury and multiligament knee injury (MLKI).

**Methods:**

A literature search using PubMed, Embase, the Cochrane Library, the Cumulative Index to Nursing and Allied Health, and Scopus from their inception to April 30, 2020 was conducted. Studies with levels I to IV evidence reporting the incidence of meniscus or cartilage injury according to early vs delayed surgery in single ACL injuries and MLKIs were included. In the meta-analysis, data based on the number of meniscus and cartilage injuries were extracted and pooled. Lysholm and Tegner scores were analyzed using two-sample Z-tests to calculate the non-weighted mean difference (NMD). A meta-regression analysis was also performed to determine the effect of single ACL injury and MLKI/study design.

**Results:**

Sixteen studies on single ACL injury and 14 studies on MLKI were included in this analysis. In the analysis, there were significant decreases in Lysholm score (NMD − 5.3 [95% confidence interval (CI) − 7.37 to − 3.23]) and Tegner score (NMD − 0.25 [95% CI − 0.45 to − 0.05]) and increases in risk of meniscus tear (odds ratio [OR] 1.73 [95% CI 1.1–2.73], *p* = 0.01) and cartilage injury (OR 2.48 [95% CI 1.46–4.2], *p* = 0.0007) in the delayed surgery group regardless of single ACL injury or MLKI. The result of the meta-regression analysis indicated that single ACL injury and MLKI/study design were not significant moderators of overall heterogeneity (*p* > 0.05).

**Conclusions:**

Our study suggests that delayed ACL surgery significantly resulted in a higher risk of meniscus tear and cartilage injury and decreased Lysholm and Tegner scores compared to early ACL surgery. The Lysholm scores in the delayed MLKI surgery group were significantly decreased, but the risks of meniscus tear and cartilage injury in the delayed MLKI surgery group remained unclear.

**Level of evidence:**

Level III, meta-analysis.

## Introduction

Treatment of multiligament knee injury (MLKI) is still challenging for orthopedic surgeons because of its complexity and severity, and it might be more common than previously reported [1–6]. The incidence of MLKI might be underestimated because of spontaneous reductions and missed diagnoses due to combined injuries, such as vascular or nerve injury [4–7]. In cases of vascular emergencies, immediate vascular repair is necessary; thus, temporal stabilization is usually applied using an external fixator [2, 5].

However, in patients without any emergencies, the timing of surgery is still controversial [4, 8–10]. Early surgical reconstruction was advocated in some previous studies [4, 6, 8], but others reported that early surgery resulted in stiffness, arthrofibrosis, and a reduced rate of return to work [1, 2, 6, 9, 11, 12]. Even in patients with an anterior cruciate ligament (ACL) injury, the timing of surgery is also controversial [13–20]. Early ACL reconstruction is likely recommended [17, 21–23] because there is an increased risk of meniscus and cartilage injury if it is delayed [21, 22]. In a study of Norwegian National Registry data, the odds of a cartilage lesion increased by nearly 1% for each month from the injury date, and the odds of cartilage lesions were nearly twice as frequent when combined with meniscal tear [24]. However, there were also studies which reported no differences between early and delayed surgery [13, 18, 25, 26]. Thus, there is still a lack of consensus regarding the timing of surgery, regardless of the type of injury (single ACL injuries or MLKI) [6, 9, 16–20].

A paucity of evidence on which to base treatment decisions and the lack of consensus in ACL injuries further complicate the management of MLKI. Questions are established to determine if the decision-making parameters are similar for knees with a single ACL injury or those with MLKI and if they provide strong outcomes.

This systemic review including meta-analysis aimed to compare clinical outcomes and incidence of concomitant injuries in patients undergoing early vs delayed surgical treatment of single ACL injury and MLKI. We hypothesized that early surgery would result in better clinical outcomes and less incidence of concomitant injuries compared to delayed surgical treatment.

## Materials and methods

### Protocol and registration

This systematic review and meta-analysis were conducted according to the Preferred Reporting Items for Systematic Reviews and Meta-Analyses (PRISMA) guidelines and using the PRISMA checklist [27] and registered using the PROSPERO International prospective register of systematic reviews [28] (CRD42020145204).

### Search strategy

A comprehensive literature search was conducted using several databases (PubMed, Embase, the Cochrane Library, the Cumulative Index to Nursing and Allied Health [CINAHL], and Scopus). In this study, the same included studies and some of the same extracted data were used in accordance with a previous meta-analysis comparing associated lesions in single ACL vs MLKIs. The date was restricted to all publications from the inception of these databases to April 30, 2020, and the search was conducted in May 2020. The search specifics were as follows: (Multiligament [All Fields] OR ((“multiple chronic conditions”[MeSH Terms] OR (“multiple”[All Fields] AND “chronic”[All Fields] AND “conditions”[All Fields] AND “acute”[All Fields]) OR “multiple chronic conditions”[All Fields] OR “multi”[All Fields]) AND (“ligaments”[MeSH Terms] OR “ligaments”[All Fields] OR “ligament”[All Fields]))) AND ((“meniscus”[MeSH Terms] OR “meniscus”[All Fields]) OR (“cartilage”[MeSH Terms] OR “cartilage”[All Fields])) AND (“knee”[MeSH Terms] OR “knee”[All Fields] OR “knee joint”[MeSH Terms] OR (“knee”[All Fields] AND “joint”[All Fields]) OR “knee joint”[All Fields]) AND ((“anterior cruciate ligament”[MeSH Terms] OR (“anterior”[All Fields] AND “cruciate”[All Fields] AND “ligament”[All Fields]) OR “anterior cruciate ligament”[All Fields] OR “acl”[All Fields]) OR “Anterior Cruciate Ligament”[Mesh] OR “anterior cruciate ligament”[All Fields]) AND AND (“chronic”[All Fields] AND “conditions”[All Fields] AND “acute”[All Fields]) AND (“1980/01/01”[PDAT]: “2020/04/30”[PDAT])) AND ((“meniscus”[MeSH Terms] OR “meniscus”[All Fields]) OR (“cartilage”[MeSH Terms] OR “cartilage”[All Fields])). The search criteria were broad to capture all potentially relevant articles, but only studies in English were included.

After combining the search results and removing duplicates, two authors independently screened the title and abstract for eligibility, and the agreement was assessed by kappa value. Subsequently, the same authors independently reviewed the full text of the selected studies. All references within the included studies were cross-referenced for inclusion if they were missed in the initial search. Systematic reviews and meta-analyses were excluded; however, their references were screened manually to find additional articles that were not identified in the first round. Disagreements were resolved by discussion between the two review authors or consultation with another author.

### Eligibility criteria

**Fig. 1:**
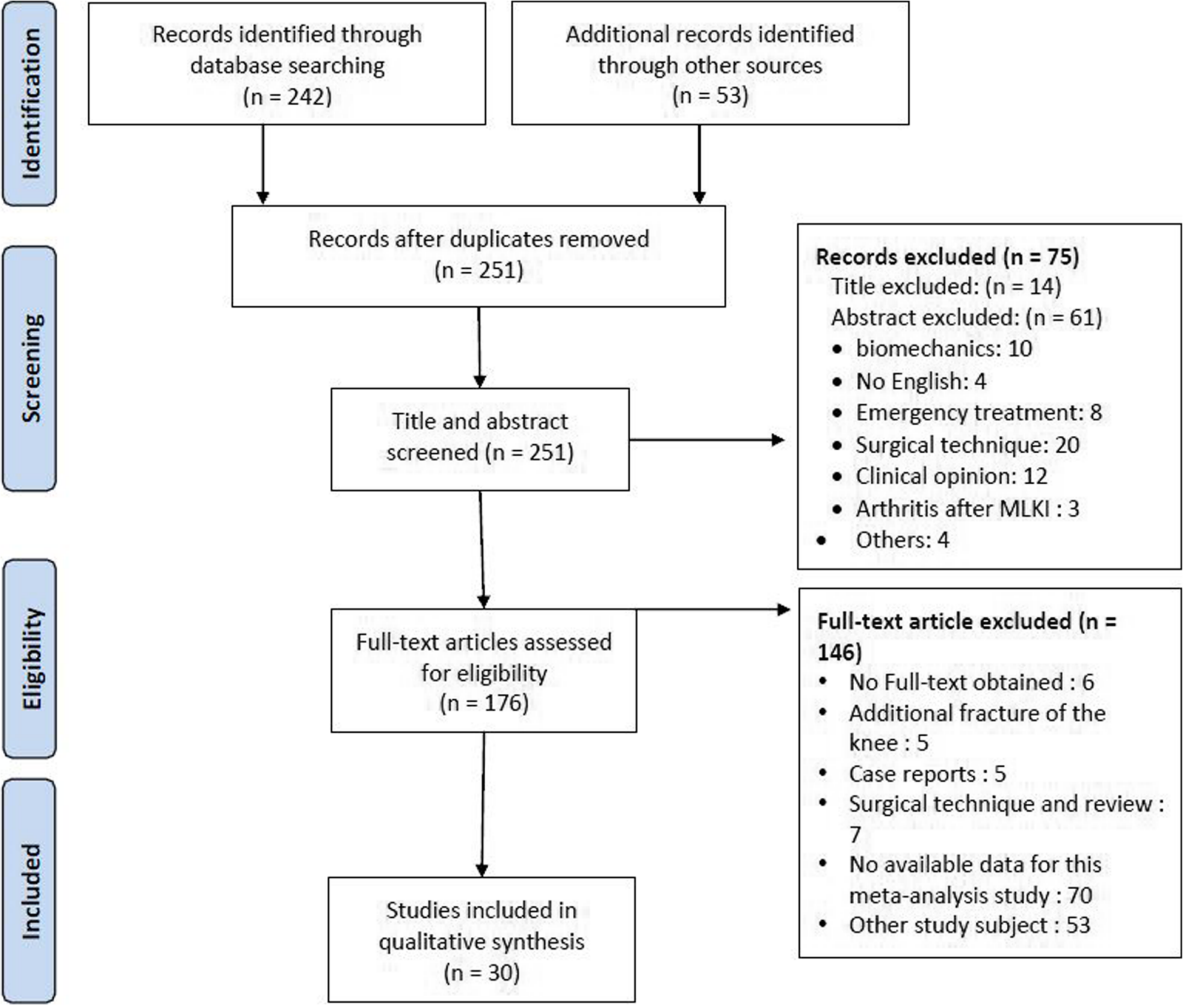
Flow diagram of articles during the selection process

Eligibility criteria for systemic review and meta-analysis were as follows: (1) English language, (2) level I to IV evidence, (3) publication between January 1980 and April 2020, (4) timing of the ligament reconstruction noted, and (5) “multiligament” defined as disruption of at least two of four major knee ligaments. The exclusion criteria were as follows: (1) not in the English language; (2) case report, clinical opinion, or technical note; (3) emergency treatment in MLKI; and (4) concomitant fracture around the knee (Fig. 1).

### Data extraction and collection

The data extraction sheet was performed based on the checklist designed by Spindler et al. [29] and the consensus of authors for variables that should be reported. The following data were extracted: (1) study type, (2) level of evidence, (3) main purpose of the study, (4) number of cases, (5) age, (6) sex, (7) combined ligament injury, (8) concomitant injuries including the meniscus, (9) cartilage injuries, (10) reported complications, (11) timing of ACL reconstruction, (12) follow-up, (13) clinical outcomes, and (14) other relevant findings including revision. The data on the timing of surgery were recorded according to the definition of each study because of the heterogeneity of the included studies. The clinical outcomes were recorded as Lysholm scores, Tegner scores, International Knee Documentation Committee (IKDC) scores, number of meniscus tears, and number of cartilage injuries. The extracted data were also cross-checked for accuracy; any disagreements were settled by discussion between the two review authors or by consultation with another author.

### Grading of the quality of evidence

The quality of evidence was evaluated by two authors using the guidelines of the Grading of Recommendations Assessment, Development and Evaluation (GRADE) working group [4]. The definitions of the grades of evidence were as follows: (1) high, when further research is unlikely to change confidence in the estimate of the effect; (2) moderate, when further research is likely to have an important impact on the confidence in the estimate of the effect and may change it; (3) low, when further research is particularly likely to have an important impact on the confidence in the estimate of the effect and is likely to change it; and (4) very low, when any estimate of the effect is extremely uncertain [4]. Disagreements were resolved by discussion and assessed by kappa value.

### Assessment of methodological quality

Two investigators independently assessed the methodological quality of each study using the Downs and Black quality assessment tool [30], which was developed for use in systemic reviews of both randomized and non-randomized studies. This tool consists of 27 questions that assess the criteria for reporting, external validity, and internal validity (measurement and confounding). The highest possible score is 32. Disagreements were resolved by discussion and assessed by kappa value. For the additional graphical assessment of the risk of bias across the studies, ROBINS-I (Risk Of Bias In Non-randomized Studies of Interventions) [31, 32] was also used, which was released by the Cochrane Non-Randomized Study Group recently.

The possibility of publication bias was examined by Egger’s test based on Galbraith plots [33] with a random-effects model. Funnel plot asymmetry and Egger’s tests were conducted to examine the possibility of publication bias. Moreover, the trim-and-fill method and calculation of a fail-safe number were also performed to evaluate the robustness of publication bias [34, 35].

### Statistical analysis

Data analyses were performed with Review Manager software (version 5.3; Nordic Cochrane Centre, the Cochrane Collaboration) and the R program (version 3.5.3, the R Foundation) using the “meta” and “metafor” packages. Statistical heterogeneity was assessed with *I*^2^ statistics: *I*^2^ > 50%, substantial heterogeneity; 20% < *I*^2^ ≤ 50%, moderate heterogeneity; *I*^2^ *<* 20%, low heterogeneity. A random-effects model was used to analyze the more robust results. Forest plots were used to show the outcome, pooled estimate of effect, and overall summary effect of each study. The treatment effects were measured by 95% confidence intervals (CIs) if the outcomes were measured on the same scale. Because of the high risk of bias due to the low level of evidence studies, the pooled mean differences were not used in clinical outcomes (Lysholm and Tegner scores). The mean synthesis and non-weighted mean differences (NMDs) of the best-evidence synthesis method were used instead to evaluate the clinical outcomes [36]. Comparisons between early and delayed values from each study were made using two-sample Z-tests using a *p* value < 0.05 (http://www.statskingdom.com/120MeanNormal2.html). The pooled odds ratio (OR) for the forest plot was also measured if the outcomes were collected as categorical data using the Mantel-Haenszel method. The heterogeneity of the binary categorical data was also evaluated using the L’Abbé plot. The subgroup analysis was performed according to the single ACL injury and MLKI groups, and meta-regression analyses with a mixed-effects model were also performed to assess the effects of the potential moderators (single ACL injury vs MLKI/prospective vs retrospective studies) on the overall heterogeneity if significant heterogeneity was observed.

The inter-rater reliability was assessed using kappa statistics (κ) to determine the degree of agreement in the study selection and risk assessment. Agreement was deemed fair (κ = 0.21–0.40), moderate (κ = 0.41–0.60), substantial (κ = 0.61–0.80), or almost perfect (κ = 0.81–1.00). In all analyses, a *p* value < 0.05 was considered significant with a two-sided tail.

## Results

### Study characteristics

The selection process for the studies is shown in the flow diagram of Fig. 1. Sixteen studies [14, 16, 23, 25, 37–48] on single ACL injury (3004 patients) and 14 studies on MLKI [30, 32, 33, 49–59] (545 patients) were included in this meta-analysis of early vs delayed surgery. Details of these included studies are presented in Table 1.

**Table 1:** Summary of the included studies

Study name	Design	Total no. of patients (criteria for early vs delayed)	Combined ligament injury	Combined meniscus injury (positive/negative)	Combined cartilage injury (positive/negative)	Clinical evaluation	Overall Follow-up
Isolated ACL injury							
Chen et al., 2015 [14, 46]	Retrospective review Cross-sectional study	293 Early: 160 cases Delayed: 133 cases (0–6 months vs longer than 7 months)	Isolated ACL injury	Early: Total: 86 cases/ MM tear: 42 cases/ LM tear: 44 cases Delayed: Total: 109 cases/MM tear: 88 cases/ LM tear: 21 cases	Early: Total: 83 cases Delayed: Total: 112 cases	Not reported	Not reported
Tandogan et al., 2004 [48]	Retrospective study	764 Early: 510 cases Delayed: 254 cases (0–12 months vs more than 12 months)	Isolated ACL injury	Total meniscus tear: 556 cases	Early: 31 cases Delayed: 55 cases	Not reported	Not reported
Manandhar et al., 2018 [40]	Prospective study	104 Early: 53 cases Delayed: 51 cases (within 3 weeks vs after 6 weeks)	Isolated ACL injury	Early: Total: 22 cases / LM tear: 12 cases / MM tear: 6 cases/ Both menisci torn: 4 cases Delayed: Total: 34 cases /LM tear: 8 cases/ MM tear: 20 cases/ Both menisci torn: 6 cases	Early: 10 cases Delayed: 28 cases	IKDC Early: 69.68 ± 8.14 vs delayed: 67.14 ± 6.08 Tegner Early 4.15 ± 1.45 vs delayed: 23.72 ± 1.34	Not reported at least 24 weeks
Meighan et al., 2003 [42]	Retrospective study	31 Early: 13 cases Delayed: 18 cases (within 2 weeks vs between 8 and 12 weeks)	Isolated ACL injury	Early: Total: 3 cases Delayed: Total: 4 cases	Not reported	Not reported	1 year
Nikolic et al., 1998 [41]	Retrospective study	182 Early: 66 cases Delayed: 65 Excluded: 51 cases (not reported, acute ACL vs ACL-deficient knee)	Isolated ACL injury	Early: Total: 51 cases Delayed: Total: 53 cases	Not reported	Not reported	Not reported
Raviraj et al., 2010 [25]	Retrospective study	99 Early: 51 cases Delayed: 48 cases (< 2 weeks vs 4–6 weeks)	Isolated ACL injury	Early: Total: 38 cases/MM tear: 18 cases/ LM tear: 20 cases Delayed: Total: 35 cases/ MM tear: 13 cases/ LM tear: 22 cases	Early: 29 cases Delayed: 31 cases	Lysholm score: early 83.1 (80–90) vs delayed 84.2 (82–90) Tegner activity score: early 6.1 (5 to 8) vs delayed 5.9 (5 to 8)	32 months (26–36)
Hur et al., 2017 [38]	Prospective study	91 Early: 48 cases Delayed: 43 cases (within 3 weeks vs more than 3 months)	Isolated ACL injury	Early: Total: 25 cases/ MM tear: 14 cases/ LM tear: 15 cases Delayed: Total: 27 cases/MM tear: 24 cases/ LM tear: 9 cases	Early: 15 cases, Delayed: 20 cases	Lysholm: Early: 94.5 ± 8.9 Delayed: 96.3 ± 3.7 Tegner: Early: 6.0 ± 1.6 Delayed: 5.6 ± 1.5	Minimum 2 years
Li et al., 2012 [39]	Retrospective study	38 Early: 17 cases Delayed: 21 cases (< 3 weeks vs ≥ 3 weeks)	Isolated ACL injury	Early: Total: 2 cases Delayed: Total: 9 cases	Early: 0 cases, Delayed: 7 cases	Lysholm: Early: 94.7 ± 9.3 Delayed: 92.2 ± 7.8 Tegner: Early: 6.6 ± 1.9 Delayed: .6.3 ± 1.3	Minimum 2 years
Ahlen and Liden, 2011 [43]	Prospective cohort study	61 Early: 30 cases Delayed: 31 cases (within 5 months vs More than 24 months)	Isolated ACL injury	Early: Total: 15 cases/ MM tear: 4 cases/ LM tear: 9 cases Delayed: Total: 20 cases/ MM tear: 14 cases/ LM tear: 2 cases	Early: 6 cases Delayed: 9 cases	Not reported	25 months (18–43)
Bottoni et al., 2008 [47]	Prospective, randomized cohort study	70 Early: 35 cases Delayed: 35 cases (within 21 days vs beyond 6 weeks)	Isolated ACL injury	Early: Total: 32 cases/MM tear: 14 cases/ LM tear: 18 cases Delayed: Total: 24 cases/MM tear: 15 cases/LM tear: 9 cases	Early: 9 cases Delayed: 5 cases	SANE: 83.1 vs 81.4 Lysholm: 80.6 vs 83.4 Tegner 5.8 vs 4.9	366 days (185–869)
Chen et al., 2015 [14, 46]	Prospective, randomized cohort study	55 Early: 27 cases Delayed: 28 cases (3–7 weeks vs 6–11 months)	Isolated ACL injury	Not reported	Not reported	Lysholm: 47.26 / 93.37 / 95.04 vs 54.1 / 91.64 / 92.64 Tegner: 2.7 / 6.3 / 6.3 vs 2.5 / 6.1 / 6.3 IKDC: 20/6/1/0 vs 17/9/2/0	61 months
Cipolla et al., 1995 [16]	Retrospective review	770 Early: 218 cases Delayed: 552 cases (within 1 week vs later in different stages)	Isolated ACL injury	Early: Total: 63 cases Delayed: Total: 412 cases	Not reported	Not reported	Not reported
Frobell et al., 2010 [45]	Prospective cohort study	121 Early: 62 cases Delayed: 59 cases (less than 10 weeks vs more than 10 weeks)	Isolated ACL injury	Early: Total 40 cases Chronic: Total 50 cases	Not reported	KOOS subscale - pain: 87.2 vs 87.7 - symptoms: 78.7 vs 83.0 - daily function: 93.5 vs 94.7 - sports function: 71.8 vs 71.2 - QOL: 67.3 vs 63.0 SF-36 - physical: 82.1 vs 78.0 - mental: 88.3 vs 83.8 Tegner: 6.5 vs 5	24.6 months (24.4–24.7) vs 25.0 months (24.7–25.2)
Frobell et al., 2013 [44]	Prospective cohort study (follow-up study of Frobell et al., 2010 [45])	121 Early: 61 cases Delayed: 59 cases (less than 10 weeks vs more than 10 weeks)	Isolated ACL injury	Early: Total 29 cases Delayed: Total 32 cases	Not reported	KOOS: 80 vs 82 KOOS subscale - pain: 91 vs 91 - symptoms: 83 vs 87 - daily function: 95 vs 97 - sports function: 76 vs 79 - QOL: 71 vs 69 SF-36 - physical: 85 vs 84 - mental: 87 vs 85 Tegner: 4 vs 4	5 years
Herbst et al., 2017 [23]	Prospective cohort study	160 Early: 51 cases Delayed: 55 cases (within 48 h vs after 6 weeks)	Isolated ACL injury	Early: MM tear: 8 cases/ LM tear: 14 cases/ Both menisci torn: 8 cases Delayed: MM tear: 13 cases/ LM tear: 13 cases/ Both menisci torn: 4 cases	Not reported	Tegner (isolated ACL): 6.7 ± 1.3 vs 6.3 ± 1.4 Tegner (with meniscus injury) 6.6 ± 1.3 vs 6.3 ± 1.5	24 months
Fok et al., 2013 [37]	Retrospective comparative study	150 Early: 97 cases Delayed: 53 cases (less than 12 months vs more than 12 months)	Isolated ACL injury	Early: Total: 58 cases Delayed: Total: 41 cases	Early: 40 cases Delayed: 25 cases	IKDC: with meniscal injury 60.4 vs without 61.3 IKDC: with chondral lesion 60.1 vs without 61.3 IKDC: Red-red tear 56.7 vs red-white tear 62.1 vs white-white tear 60.1 Tegner score: preop (< 12 months) 3.92 vs preop (> 12 months 3.41)	Not reported
Multiligament injuries							
Krych et al., 2015 [58]	Retrospective study	122 Early: 62 cases Delayed: 60 cases (within 3 months vs after 3 months)	KD I: 25 KD II: KD III: 72 KD IV: 16	Early: 19 cases Delayed: 21 cases	Total cartilage injury 52 (48.0%) / 70 (52.0%)	Not reported	Not reported
Tardy et al., 2017 [56]	Retrospective study	39 Early: 22 cases Delayed: 17 cases (7–30 days vs after 3 months)	PMC vs PLC injury	Early: 6 cases Delayed: 7 cases	Not reported	Objective IKDC: PMC 2A, 16B,1C vs PLC 1A,13B,6C” Subjective IKDC: PMC 81 ± 15 vs PLC 70 ± 17″ Lysholm PMC 89 ± 7 vs PLC 79 ± 11 Sports activity level PMC: 8 at same level /10 decreased in activity level/1 stopped sports PLC: 4 at same level/10 decreased in activity level/ 6 stopped sports	57 months (12–129)
Moatshe et al., 2017 [59]	Prospective cohort study	65 Early: 33 cases Delayed: 32 cases (less than 21 days vs more than 21 days)	KD I: 0 KD II: 4 KD III: 55 KD IV: 6	25 (38.5%) / 40 (61.5%)	25 (38.5%) / 40 (61.5%)	Lysholm score Early: 86.9 ± 15 Delayed: 81 ± 19 Tegner activity Early: 4 ± 1.8/ Delayed: 4 ± 2 KOOS symptoms 78 KOOS pain 81 KOOS ADL 87 KOOS sport 54 KOOS QOL 64 Single leg hop test 88–93% of the uninjured leg	13.1 years (10–18.8 )
Li et al., 2013 [51]	Retrospective study	15 Early: 6 cases Delayed: 9 cases (< 3 weeks vs ≥3 weeks)	KD I: 0 KD II: 7 KD III: 8 KD IV: 0	Not reported	Not reported	Lysholm score Early: 89.4 ± 4.4 Delayed: 82.1 ± 6.3 Tegner activity Early: 3.9 / Delayed: 3.4	7.5 years (6–12)
Liow et al., 2003 [8]	Retrospective study	22 Early: 8 cases Delayed: 14 cases (< 2 weeks vs ≥2 weeks)	KD I: 7 KD II: 2 KD III: 11 KD IV: 0	Not reported	Not reported	Lysholm score Early: 87 (81–91) Delayed: 75 (53–100) Tegner activity Early: 5 / Delayed: 4.4	32 months (11–77)
Noyes et al., 1997 [57]	Retrospective study	11 Early: 7 cases Delayed: 4 cases (Early: mean 14 days Delayed: mean 22 months)	Both cruciate ligaments torn: 10, All ligaments torn: 1 case	Not reported	Early: 0 Delayed: 3	Not reported	4.5 years
Subbiah et al., 2011 [50]	Retrospective study	19 Early: 11 cases Delayed: 8 cases (< 3 weeks vs ≥3 weeks)	KD I: 5 KD II: 3 KD III: 11 KD IV: 0	Total meniscus injury 16 (84%)/3 (16%)	Not reported	Lysholm score Early: 93.3 ± 6.6 Delayed: 90 ± 5.8	22 months (14–33)
Wascher et al., 1999 [49]	Retrospective study	13 Early: 9 cases Delayed: 4 cases (< 3 weeks vs ≥3 weeks)	ACL/PCL/MCL: 7 (53.8%) ACL/PCL/PLC: 6 (46.2%)	Early: Total: 4 cases Delayed: Total: 2 cases	Not reported	Lysholm score Early: 91.8 ± 7.1 Delayed: 79.3 ± 22.7 IKDC: 6 nearly normal (46.2%) Meyers: 11 excellent or good (84.6%)	38 months
Zhang et al., 2013 [52]	Retrospective study	59 Early: 48 cases Delayed: 11 cases (< 3 weeks vs > 3 weeks)	ACL/PCL/MCL/PLC	Not reported	Not reported	Lysholm score Early: 87.6 ± 10.2 Delayed: 80.5 ± 13.3	2.5 years
Tzurbakis et al., 2006 [10]	Retrospective study	48 Early: 38 cases Delayed: 10 cases (within 3 weeks vs more than 3 weeks)	Group A (ACL + medial): 12 (25%) Group B (cruciate + PLC): 11 (22.9%) Group C (bicruciate + collateral): 25 (52.1%)	Total meniscus tear MM: 20 (41.7%) / 28 (58.3%) LM: 13 (27.1%) / 35 (72.9%)	Total cartilage injury 6 (12.5%) / 42 (87.5%)	Tegner: Early: 4.4 ± 2.1 Delayed: 5.2 ± 2.2 Lysholm: Early: 87 ± 12.3 Delayed: 81.7 ± 13.3	51.3 ± 29.9 months (24–96)
Harner et al., 2004 [9]	Retrospective cohort study	31 Early: 19 cases Delayed: 12 cases (within 3 weeks vs more than 3 weeks)	ACL/PCL/MCL/PLC	Not reported	Not reported	Lysholm: Early: 91 Delayed: 80 KOOS (daily): Early: 91 vs Delayed: 84 KOOS (sports): Early: 89 vs Delayed: 69 Meyers: Early: 16/19 vs Delayed: 7/12	44 M / minimum 2Y
Owens et al., 2007 [54]	Retrospective study	28 Early: 20 cases Delayed: 8 cases (within 14 days vs greater than 14 days)	ACL/PCL/MCL/PLC	Total meniscus injury 14 (50%) / 14 (50%)	Not reported	Lysholm score Early: 91.2 ± 6.52 Delayed: 83.6 ± 7.3	48 months (13–82)
Fanelli et al., 1996 [55]	Retrospective study	21 Early: 13 cases Delayed: 8 cases (2–4 weeks vs 6 months–16 years)	All were PCL/PLC injuries	Not reported	Not reported	Lysholm score Early: 91.2 / Delayed: 91.6 Tegner: Early 5.2 / Delayed: 5.0	Minimum 24 months (24–54)
Wajsfisz et al., 2014 [53]	Retrospective study	53 Early: 10 cases Delayed: 43 cases (within 21 days vs more than 21 days)	Not reported	Not reported	Not reported	Lysholm score Early: 83 / Delayed: 76.5	49 months (12–146)

*ACL* anterior cruciate ligament, *MM* medial meniscus, *LM* lateral meniscus, *IKDC* International Knee Documentation Committee, *KOOS* Knee Injury and Osteoarthritis Outcome Score, *QOL* quality of life, *KD* Schenck knee dislocation type, *PMC* posteromedial corner, *PLC* posterolateral corner, *ADL* activities of daily living, *PCL* posterior cruciate ligament, *MCL* medial collateral ligament

### Assessment of methodological quality

**Fig. 2:**
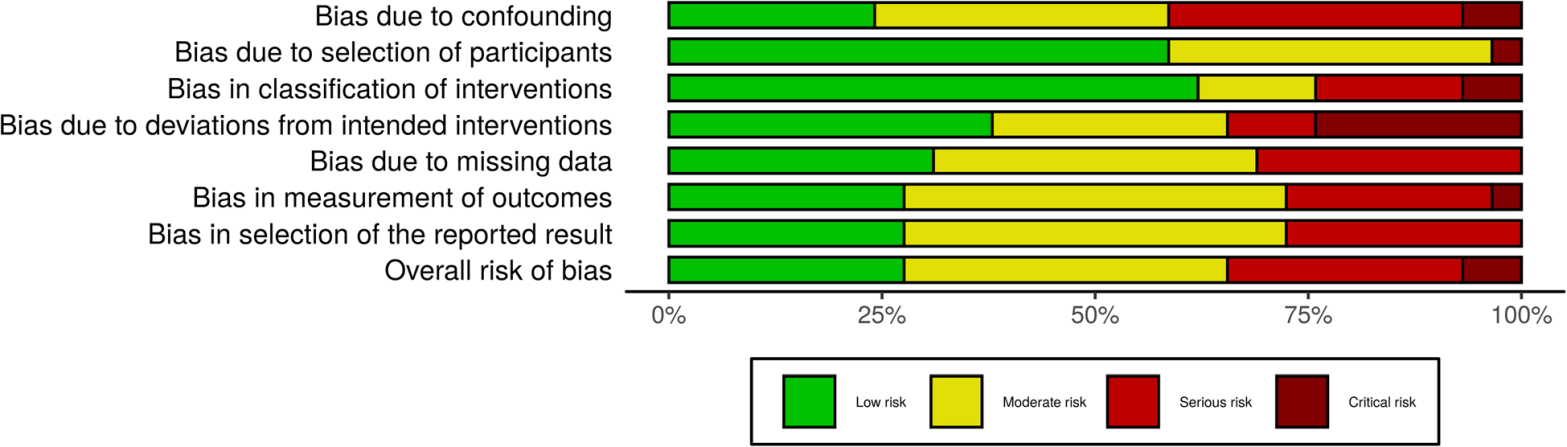
Bar plot for risk of bias using ROBINS-I tool

The results of the quality assessment in the included studies are shown in Table 2 (κ = 0.73, substantial agreement). The overall bar plot of the ROBINS-I tool is summarized in Fig. 2 and Additional file 1: Figure S1 (κ = 0.83, almost perfect agreement).

**Table 2:** Quality assessment of included studies for meta-analysis

Study name	Design	Total no. of patients	Reporting (11)	External validity	Internal validity: bias	Internal validity: confounding (selection bias)	Power	Total
Isolated ACL injury								
Chen et al., 2015 [14, 46]	Retrospective study	293	5	1	3	3	5	17
Tandogan et al., 2004 [48]	Retrospective study	764	6	1	3	2	5	17
Manandhar et al., 2018 [40]	Prospective cohort study	104	9	1	5	3	5	23
Meighan et al., 2003 [42]	Retrospective study	31	7	1	3	3	2	16
Nikolic et al., 1998 [41]	Retrospective study	182	7	1	3	2	5	18
Raviraj et al., 2010 [25]	Retrospective study	99	8	1	3	3	4	19
Hur et al., 2017 [38]	Prospective study	91	8	1	3	4	5	21
Li et al., 2012 [39]	Retrospective study	38	7	1	3	3	4	18
Ahlen and Liden et al., 2011 [43]	Prospective cohort study	61	8	1	3	3	5	20
Bottoni et al., 2008 [47]	Prospective, randomized study	70	10	1	4	6	4	25
Chen et al., 2015 [14, 46]	Prospective, randomized study	55	8	1	4	4	3	20
Cipolla et al., 1995 [16]	Retrospective study	1103	3	1	2	2	5	13
Frobell et al., 2010 [45]	Prospective cohort study	121	10	1	5	4	5	25
Frobell et al., 2013 [44]	Prospective cohort study	121	10	1	5	4	5	25
Herbst et al., 2017 [23]	Prospective cohort study	160	8	1	5	3	5	22
Fok et al., 2013 [37]	Retrospective comparative study	150	10	1	3	2	4	20
Multiligament injuries								
Krych et al., 2015 [58]	Retrospective study	122	3	1	3	3	5	15
Tardy et al., 2017 [56]	Retrospective study	39	4	1	3	3	2	13
Moatshe et al., 2017 [59]	Prospective cohort study	65	9	1	4	3	4	21
Li et al., 2013 [51]	Retrospective study	15	4	1	3	3	0	11
Liow et al., 2003 [8]	Retrospective study	22	5	1	3	3	1	13
Subbiah et al., 2011 [50]	Retrospective study	19	7	1	3	2	0	13
Zhang et al., 2013 [52]	Retrospective study	59	7	1	3	2	3	16
Tzurbakis et al., 2006 [10]	Retrospective study	48	5	1	2	0	3	11
Noyes, et al., 1997 [57]	Retrospective study	11	3	1	2	0	0	6
Harner et al., 2004 [9]	Retrospective study	31	9	1	3	3	2	18
Owens et al., 2007 [54]	Retrospective study	28	4	1	2	0	1	8
Wascher et al., 1999 [49]	Retrospective study	13	5	1	2	0	0	8
Fanelli et al., 1996 [55]	Retrospective study	21	3	0	1	0	1	5
Wajsfisz et al., 2014 [53]	Retrospective study	53	4	0	0	0	0	4

A funnel plot was used to evaluate the scores which could be obtained by weighted values (total meniscus tears and cartilage injuries). The funnel plot suggested a publication bias in the assessment of meniscus tear; the trim-and-fill method and calculation of fail-safe number were then performed to further assess the publication bias.

**Fig. 3:**
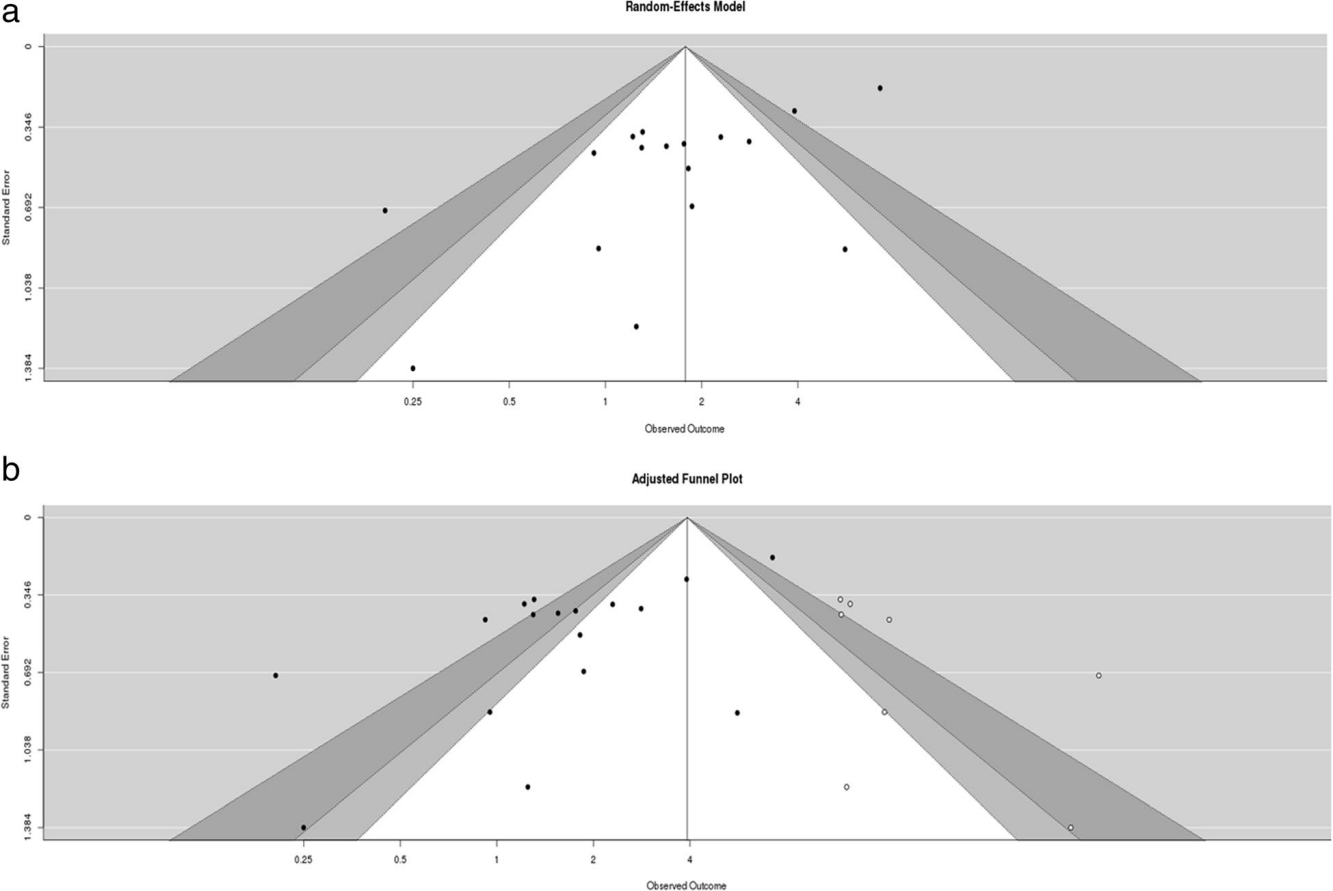
Funnel plot for risk of meniscus tear. **a** Evidence of asymmetry was observed (*p* = 0.000). **b** Adjusted funnel plot after the trim-and-fill method indicated the absence of publication bias, and the observed outcome was changed to reinforce direction of the outcome

In the analysis of meniscus tear, evidence of asymmetry was observed (Fig. 3a), and this result was further supported by an analysis using Egger’s test (*p* = 0.000). The adjusted funnel plot after the trim-and-fill method (Fig. 3b) indicated the absence of publication bias with eight added studies, but the observed outcome was changed to reinforce the direction of the outcome (before vs after trim and fill, OR 1.73 vs 3.42). Moreover, the fail-safe number was calculated by the Rosenthal approach [34] as 227 (*p* < 0.0001), which is a robust result for publication bias for this study.

In the analysis of cartilage injury, there was no evidence of publication bias (*p* = 0.618, Additional file 2: Figure S2). The fail-safe number was calculated as 168 for the cartilage injury (*p* < 0.0001). The cartilage injury analysis showed a robust result for publication bias.

### GRADE evidence quality of each outcome

The GRADE evidence quality of each outcome is presented in Table 3 (κ = 0.65, substantial agreement). Four outcomes were separately evaluated: one of very low quality and three of low quality. The overall results were found to have a trend of increased risk of meniscus tear and cartilage injury and decreased Lysholm and Tegner scores in the delayed surgery group. However, almost all outcomes had limitations in study design, imprecise data, and directness of the studies.

**Table 3:** GRADE evidence quality for each outcome

Quality assessment						Summary of findings			
Number of studies	Design	Quality	Consistency	Directness	Other modifying factors	No. of patients		Summary	Quality
						Delayed	Early		
Concomitant meniscus tear									
18	RCT: 7 Non-RCT: 11	Very serious limitations (−2)	Important inconsistency (−1)	Some uncertainty (−1)	Evidence of a dose-response gradient (+ 1)	1308	1062	The incidence of meniscus tear in delayed group was higher than in early group. Only 3 studies reported higher incidence of meniscus tear in early group	Low
Lysholm score									
17	RCT: 4 Non-RCT: 13	Very serious limitations (−2)	No important inconsistency	Some uncertainty (−1)	Imprecise or sparse data (− 1). Evidence of a dose-response gradient (+ 1)	402	455	The Lysholm scores decreased in delayed surgery group. Only 2 studies reported higher scores in delayed surgery group	Low
Tegner score									
15	RCT: 8 Non-RCT: 7	Very serious limitations (−2)	No important inconsistency	Some uncertainty (−1)	None	496	524	The Tegner scores decreased in delayed surgery group, but those for delayed surgery group in MLKI were marginal	Low
Concomitant cartilage injury									
10	RCT: 5 Non-RCT: 5	Very serious limitations (−2)	Important inconsistency (−1)	Some uncertainty (− 1)	Imprecise or sparse data (− 1)	673	1008	The incidence of cartilage injury in delayed group was higher than in early group. Only 1 study of MLKI was included for this meta-analysis.	Very low

*GRADE* Grading of Recommendations Assessment, Development and Evaluation, *RCT* randomized controlled trial, *MLKI* multiligament knee injury

### Risk of concomitant meniscus tear

**Fig. 4:**
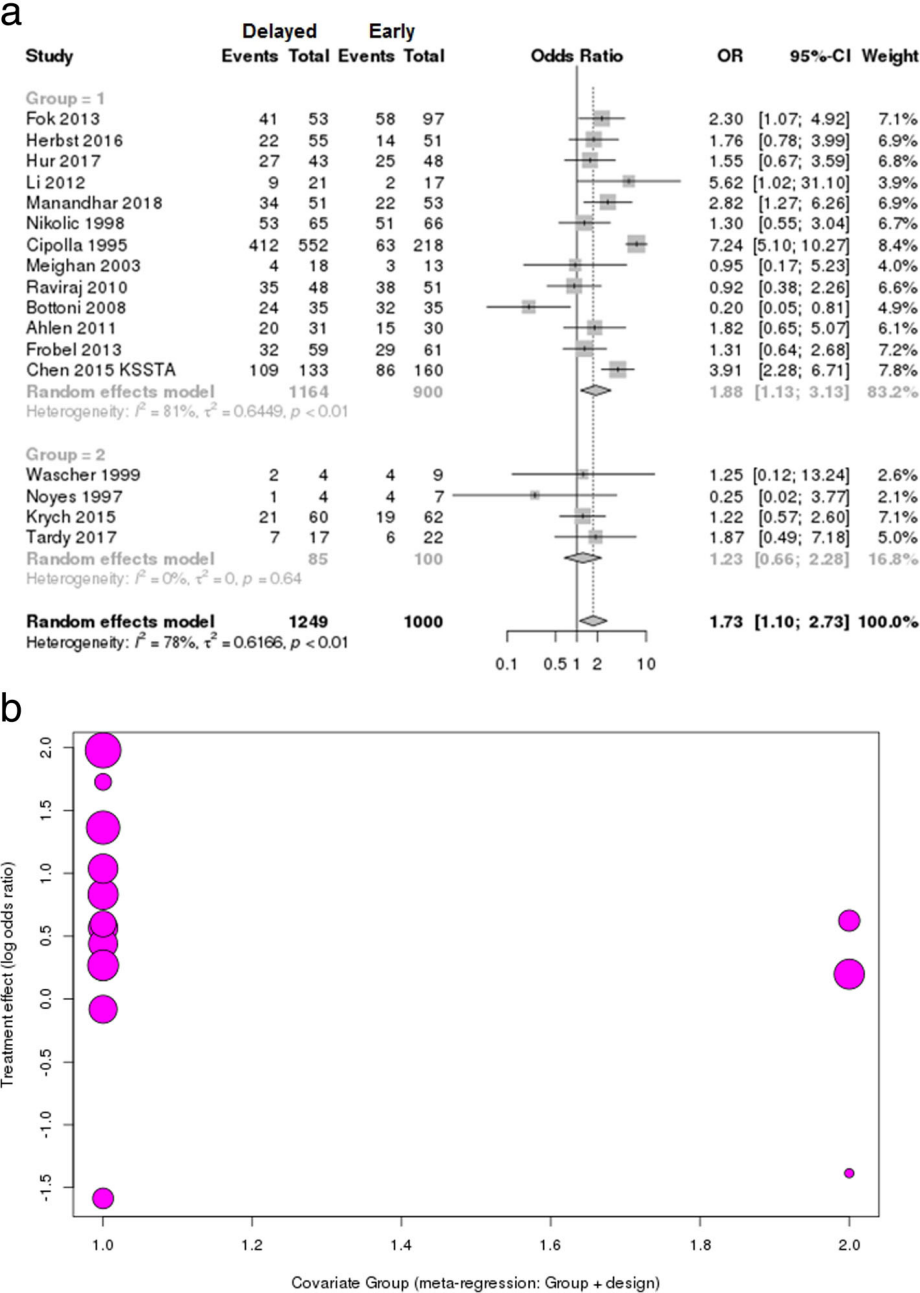
**a** Forest plot of odds ratio with 95% confidence intervals in meniscus tear. The *gray squares* represent the results of each study. *Ends of the horizontal bars* represent 95% confidence intervals. *Dark gray diamonds* show the overall results of all studies. Random-effects models were used. **b** Plot of the meta-regression analysis indicated that single ACL injury and MLKI/prospective and retrospective design were not significant moderators of overall heterogeneity (*p* = 0.255)

The number of total meniscus injuries according to early vs delayed surgery was determined in 13 studies (2064 patients) on single ACL injury [23, 25, 37–44, 46, 47] and 4 studies (185 patients) on MLKI [49, 56–58]. The overall risk of meniscus tear in the delayed surgery group was significantly higher than that in the early surgery group, but this showed substantial heterogeneity (OR 1.73 [95% CI 1.1–2.73], *p* = 0.01; *I*^2^ = 78%, *p* < 0.01), as did the L’Abbé plot (Fig. 4a, Additional file 3: Figure S3). In the subgroup analysis according to single ACL injury and MLKI, the risk of meniscus tear in delayed surgery of single ACL injury was significantly higher than that in early surgery with substantial heterogeneity (OR 1.88 [95% CI 1.13–3.13], *p* = 0.015; *I*^2^ = 81%, *p* < 0.01), but the risk of meniscus tear in MLKI was not significant regardless of the timing of surgery (early or delayed) (OR 1.23 [95% CI 0.66–2.28], *p* = 0.512; *I*^2^ = 0%, *p* = 0.64) (Fig. 4a).

The result of the meta-regression analysis indicated that single ACL injury and MLKI/prospective and retrospective design were not significant moderators of overall heterogeneity (Fig. 4b, *p* = 0.255).

### Risk of concomitant cartilage injury

**Fig. 5:**
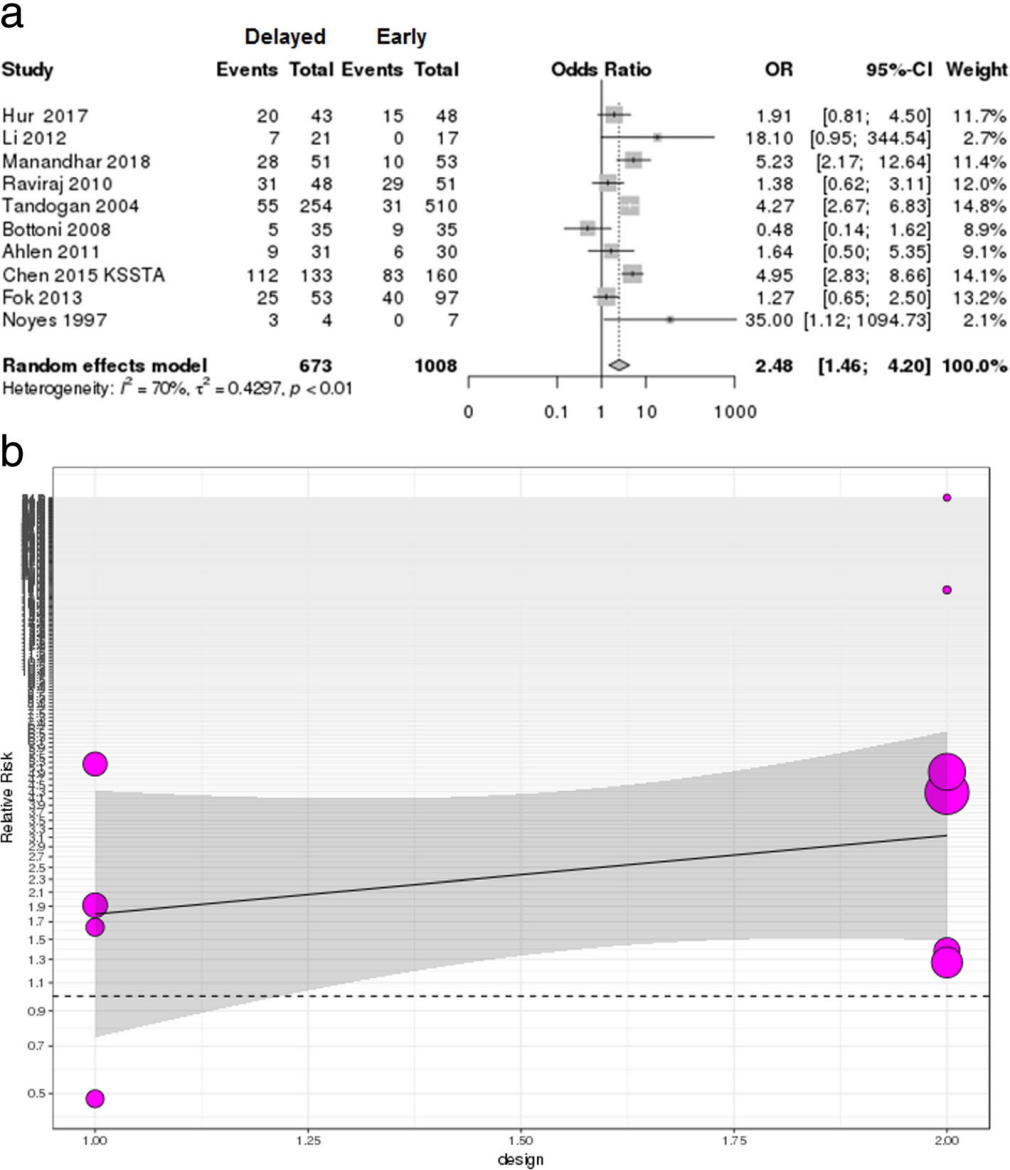
**a** Forest plot of odds ratio with 95% confidence intervals in cartilage injury. The *gray squares* represent the results of each study. *Ends of the horizontal bars* represent 95% confidence intervals. *Dark gray diamonds* show the overall results of all studies. Random-effects models were used. **b** Plot of the meta-regression analysis indicated that prospective and retrospective design were not significant moderators of overall heterogeneity (*p* = 0.336)

The number of cartilage injuries according to the timing of surgery (early vs delayed) was described in 10 studies (1681 patients) regardless of single ACL injury or MLKI [25, 37–40, 43, 46–48, 57]. Only one study [57] on MLKI reported cartilage injury result according to the timing of surgery (early vs delayed); thus, the subgroup analysis was not performed. The overall risk of cartilage injury in the delayed surgery group was significantly higher than that in the early surgery group, but this showed substantial heterogeneity (OR 2.48 [95% CI 1.46–4.2], *p* = 0.0007; *I*^2^ = 70%, *p* < 0.01), as did the L’Abbé plot (Fig. 5a, Additional file 4: Figure S4).

The result of the meta-regression analysis indicated that prospective and retrospective design were not significant moderators of overall heterogeneity (Fig. 5b, *p* = 0.336).

### Lysholm score

**Fig. 6:**
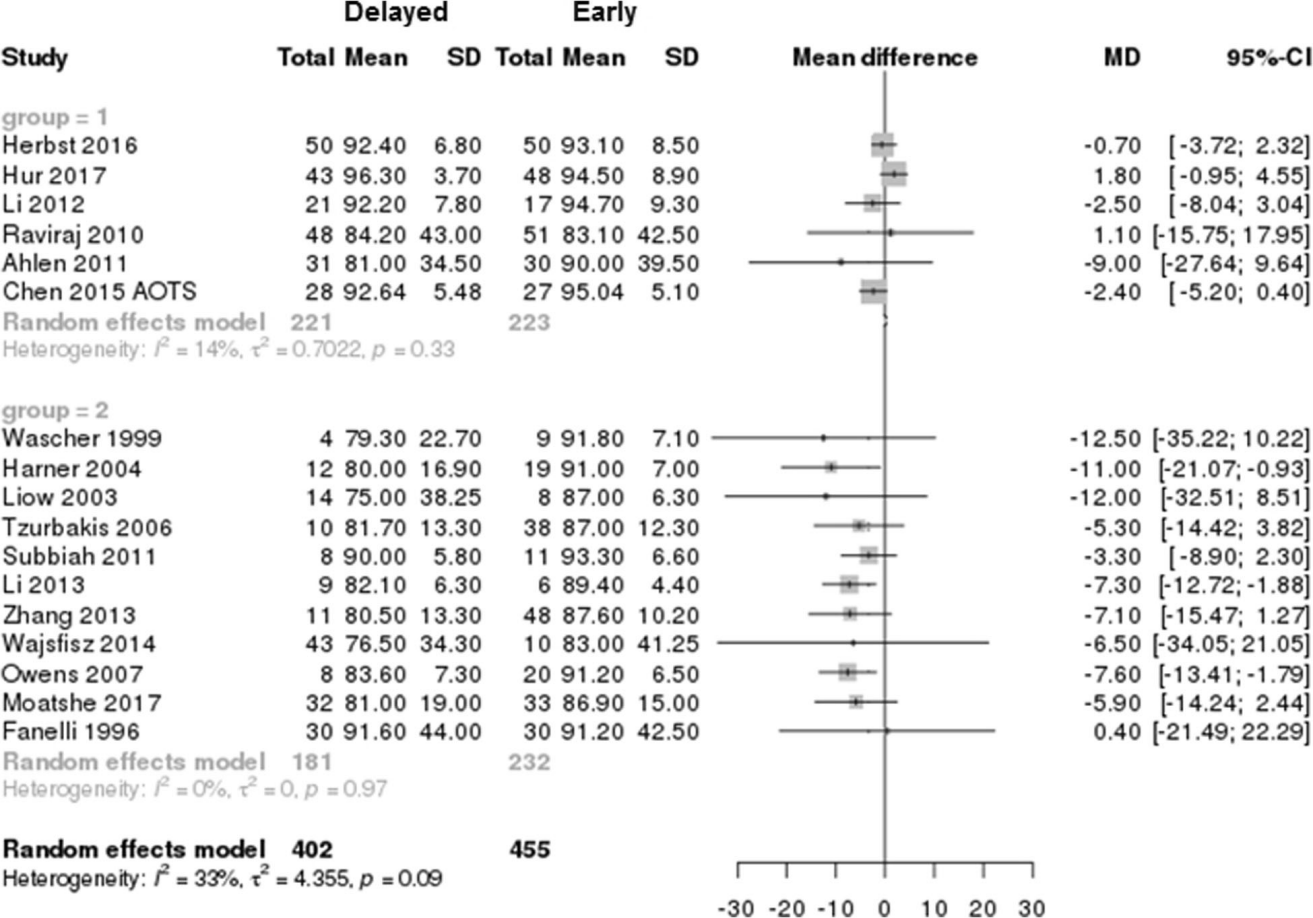
Forest plot of mean difference with 95% confidence intervals in Lysholm scores. The *gray squares* represent the results of each study. *Ends of the horizontal bars* represent 95% confidence intervals. Due to the heterogeneity, the non-weighted mean differences were used to assess overall results by the best-evidence synthesis; they were not shown in this plot

The Lysholm scores according to the timing of surgery (early vs delayed) were described in 6 studies (444 patients) on single ACL injury [23, 25, 38, 39, 43] and 11 studies (413 patients) on MLKI [30, 32, 33, 49–55, 59]. The overall Lysholm scores in the delayed surgery group were lower than those in the early surgery group (early vs delayed, 89.9 ± 3.64 vs 85.3 ± 5.9; *p* < 0.001; NMD − 5.3 [95% CI − 7.37 to − 3.23]) (Fig. 6). In the subgroup analysis according to the type of injury (single ACL injury and MLKI), the Lysholm score of the delayed surgery MLKI group was significantly lower than that of the early surgery MLKI group (early vs delayed, 89.0 ± 2.87 vs 82.8 ± 4.61; *p* < 0.001; NMD − 7.1 [95% CI − 9.24 to − 4.96]), and the Lysholm score of the delayed surgery single ACL group was also significantly lower than that of the early surgery single ACL group (early vs delayed, 91.7 ± 4.21 vs 89.8 ± 5.34; *p* < 0.001; NMD − 1.95 [95% CI − 4.78 to 0.88]).

### Tegner score

**Fig. 7:**
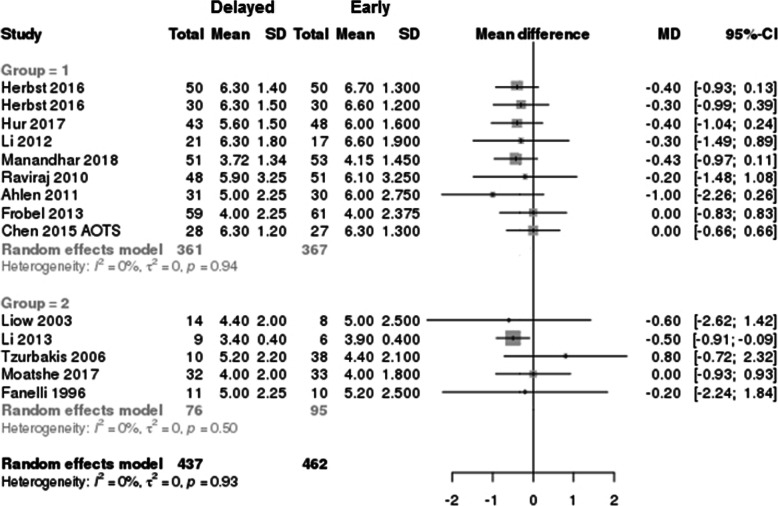
Forest plot of mean difference with 95% confidence intervals in Tegner scores. The *gray squares* represent the results of each study. *Ends of the horizontal bars* represent 95% confidence intervals. Due to the heterogeneity, the non-weighted mean differences were used to assess overall results by the best-evidence synthesis; they were not shown in this plot

The Tegner scores according to the timing of surgery (early vs delayed) were described in 9 studies (728 patients) on single ACL [23, 25, 38–40, 43, 44] and five studies (171 patients) on MLKI [30, 33, 51, 55, 59]. In the study by Herbst et al. [23], the researchers reported the results as separate groups according to the meniscus injury; thus, we analyzed the results as two different studies. The overall Tegner scores in the delayed surgery group were significantly decreased (early vs delayed, 5.4 ± 1.05 vs 5.1 ± 1.01; *p* < 0.001; NMD − 0.25 [95% CI − 0.45 to − 0.05]) compared to those in the early surgery group (Fig. 7). In the subgroup analysis according to the type of injury (single ACL injury and MLKI), the Tegner score of the delayed surgery single ACL injury group was significantly lower than that of the early surgery single ACL injury group (early vs delayed, 5.8 ± 0.97 vs 5.5 ± 0.97; *p* < 0.001; NMD − 0.3 [95% CI − 0.51 to − 0.15]), but the Tegner score in the MLKI group was not significant, regardless of the timing of surgery (early vs delayed, 4.5 ± 0.52 vs 4.4 ± 0.66; *p* = 0.28, NMD − 0.1 [95% CI − 0.54 to 0.34]).

### Sensitivity analysis

We performed a meta-analysis with the results of meniscus tear and cartilage injury, after removing a single study, for the sensitivity analysis (Additional file 5: Figure S5 and Additional file 6: Figure S6). The results of the sensitivity analysis were similar to those of the initial analysis.

## Discussion

The most important finding of this meta-analysis was that the delayed ligament surgery group was significantly found to have a higher risk of meniscus tear and cartilage injury and decreased Lysholm and Tegner scores compared to the early ligament surgery group. In single ACL injury, a high risk of meniscus tear and decreased Lysholm and Tegner scores were found in the delayed surgery group. In MLKI, only the Lysholm score was decreased in the delayed surgery group. However, the high risk of bias due to the low level of evidence studies was also affected by the results of clinical outcomes (Lysholm and Tegner scores), and the clinical relevances of these results are still questionable.

Several previous systemic reviews and meta-analyses reported that the timing of the ACL reconstruction would not affect the outcomes [34, 44, 45], but other meta-analyses with high levels of evidence reported similar but somewhat superior results for early ACL reconstruction compared to delayed surgery [25, 60]. Recent studies, other than meta-analyses, reported that early ACL reconstruction showed better clinical results due to rapid restoration of stability and function and less risk of meniscus and cartilage injury than delayed ACL reconstruction [24, 41, 42, 61, 62]. In a recent meta-analysis of MLKI [4], early ligament reconstruction was recommended because of superior patient-reported and clinical outcomes. Of all patients undergoing early surgery, 31% had a normal or near-normal knee, compared to only 15% of patients undergoing delayed reconstruction [4]. However, Mook et al. [1] found worse outcomes in terms of stiffness, anterior stability, and clinical outcomes in the early surgery group.

The studies on MLKI are extremely heterogeneous, so the results might change according to the inclusion criteria. Nonetheless, all relevant articles were focused on the clinical or stability outcomes, not the incidence of meniscus tear and cartilage injury in early vs delayed surgery and the differences between single ACL injury and MLKI. Although Ferguson et al. [20] performed a meta-analysis on ACL injuries, including meniscus tear and cartilage injury, only six studies with various study designs were included. Hohmann et al. [11] performed a meta-analysis on MLKI but did not assess the outcomes of meniscus or cartilage injury and comparison with single ACL injury, which could be a control group with well-known results. Thus, it was useful to perform a meta-analysis and systemic review with studies including meniscus tear and cartilage injury results to assess the differences between early and delayed ligament reconstructions and the subgroup differences between MLKI and single ACL injury.

This systemic review including meta-analysis revealed that early ligament reconstruction could result in better Lysholm and Tegner scores and lesser risk of meniscus and cartilage injury in overall ligament injuries. The overall risk of meniscus tear and cartilage injury in the delayed surgery group was significantly increased compared to that in the early surgery group (meniscus, OR 1.73, *p* = 0.015; cartilage, OR 2.48, *p* = 0.0007), and this trend was found mostly in patients with a single ACL injury (Figs. 4a and 5a). These results are similar to those in studies on ACL injury indicating that chronic ACL injuries increase the risk of meniscus and cartilage injuries, which were recognized as predictors of osteoarthritis in the long-term follow-up [24, 41, 42, 61–63].

However, there were also studies that showed no differences between early and delayed surgeries [34, 44, 45]. In the recent meta-analysis by Ferguson et al. [20], the risk of meniscus tear or cartilage injury was not significant between the early and delayed surgery groups, although the result for cartilage injury was borderline significant (*p* = 0.06). This difference might originate from the difference in the number of included studies due to the longer inclusion period of this study. Moreover, the Tegner scores in the meta-analysis by Ferguson et al. [20] were 0.39 point greater in the early surgery group than in the delayed surgery group, which was similar to the results of our analysis (− 0.25 [95% CI − 0.45 to − 0.05]). Although these observed scores are questionable in clinical relevance, one should address that delayed ACL surgery might have lower functional outcomes. Thus, while there were no differences in observed meniscal/chondral lesions and small differences in observed Tegner scores between the early or delayed surgery groups in previous studies [34, 45, 64], based on the results and wider included literature in this study, early intervention would be recommended to decrease the risk of developing meniscal/cartilage lesions and potentially reduce the subsequent risk of osteoarthritis and low functional outcomes.

In MLKI, the timing of surgery did not significantly affect the incidence of meniscus tears and postoperative Tegner scores. The Lysholm scores were higher in the early surgery group than in the delayed surgery group (Fig. 6). Because all published studies on MLKI had low levels of evidence in study designs and showed heterogeneity, it is possible that future publications may change the trend of this meta-analysis by either confirming the outcome of this analysis or reversing these observed outcomes. Despite the limitations of the included study, the results of this meta-analysis are also in line with those of previous studies, which showed favorable results for early surgery [4, 30, 32, 33, 49, 65]. According to Levy [65] and Hohmann [11], higher Lysholm and IKDC scores and satisfactory final range of motion (ROM) were found in the early surgery group. McKee et al. [66] and Vicenti et al. [60] also suggested the general consensus and results of early surgery in MLKI, within the first 3 weeks, and found greater ROM in the early surgery group than in the delayed surgery group [60]. The results of our analysis, including the Lysholm score, were similar to those of previous studies [4, 65]. Although other previous studies reported that a high incidence of arthrofibrosis was found in the early surgery group [26, 67–69], and good clinical outcomes were found in the delayed surgery group [12, 55, 70, 71], the results of this analysis and previous systemic reviews and meta-analyses [4, 60, 65, 66] suggest that early surgery of MLKI yields higher Lysholm scores with similar incidence of concomitant injuries and improved functional outcomes compared to delayed surgery.

### Limitations

This study has some limitations. First, the standard of timing was different according to the studies: the definition of early ranged broadly up to 5 months and that of delayed ranged from 10 weeks to 24 months. Therefore, we included studies based on the author’s definition of early and delayed rather than separate them as arbitrary time frames of early and delayed reconstructions by another definition. Second, all studies were included in the meta-analysis even if they did not report early vs delayed surgery as a primary outcome, especially in studies on MLKI; thus, the results were found to have substantial heterogeneity. It is extremely difficult to evaluate the clinical results on MLKI as prospective, comparative studies because of the heterogeneous nature of the injuries. Thus, we included all possible outcomes in this study and compared the results of single ACL injury as a control group. Third, relatively few studies with lower levels of evidence and small sample sizes were also major limitations, especially in studies on MLKI. Due to the searching strategy, the studies including early vs delayed and meniscus/cartilage injuries were selected for the systemic review, and the number of studies seems to be low. Moreover, in MLKIs, the different injury mechanisms and irregular knee ligament involvements and any possible combined traumas (vascular, nerve, fractures, etc.) contribute to the difficulties of analysis and obtaining consensus of treatment. The high risk of bias and heterogeneous publication of low level of evidence studies make the pooling results difficult to interpret as it is. However, in the meta-regression analysis according to the study design, there was no significant effect of the heterogeneity (Figs. 4b and 5b). Thus, we focused on the prevalence of concomitant injuries in the meta-analysis rather than the clinical outcomes, although the clinical outcomes were also reported as non-weighted means.

## Conclusions

Our study suggests that delayed ACL surgery significantly resulted in higher risk of meniscus tear and cartilage injury and decreased Lysholm and Tegner scores compared to early ACL surgery. The Lysholm scores in the delayed MLKI surgery group were significantly decreased, but the risks of meniscus tear and cartilage injury in the delayed MLKI surgery group remained unclear.

## Supplementary Information

**Additional file 1: Figure S1.** Traffic light plot for risk of bias using ROBINS-I tool.

**Additional file 2: Figure S2.** Funnel plot for cartilage injury. No evidence of asymmetry was observed (*p* = 0.618).

**Additional file 3: Figure S3.** L’Abbé plot of meniscal tear indicated moderate heterogeneity among the included studies.

**Additional file 4: Figure S4.** L’Abbé plot of cartilage injury indicated moderate heterogeneity among the included studies.

**Additional file 5: Figure S5.** Forest plot for sensitivity analysis of the risk of meniscus tear. The results were also significant, similar to those in the initial assessment.

**Additional file 6: Figure S6.** Forest plot for sensitivity analysis of the risk of cartilage injury. The results were also significant, similar to those in the initial assessment.

## Data Availability

The datasets used and/or analyzed during the current study are available from the corresponding author on reasonable request.
