# Supplementary figures and images for: A systematic review comparing the results of early vs delayed ligament surgeries in single anterior cruciate ligament and multiligament knee injuries
Source: Knee Surg Relat Res. 2021 Jan 7;33:1. doi: 10.1186/s43019-020-00086-9 (PMC7792064; doi:10.1186/s43019-020-00086-9)

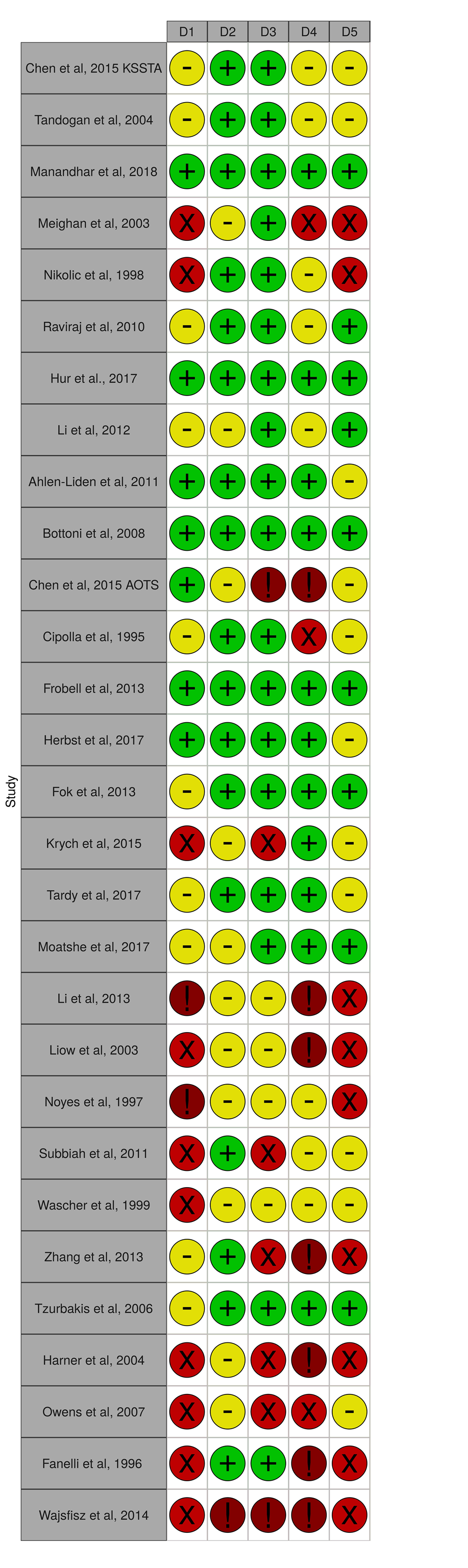

Supplement: Supplementary file 1 — Additional file 1: Figure S1. Traffic light plot for risk of bias using ROBINS-I tool. [file 43019_2020_86_MOESM1_ESM.tif]

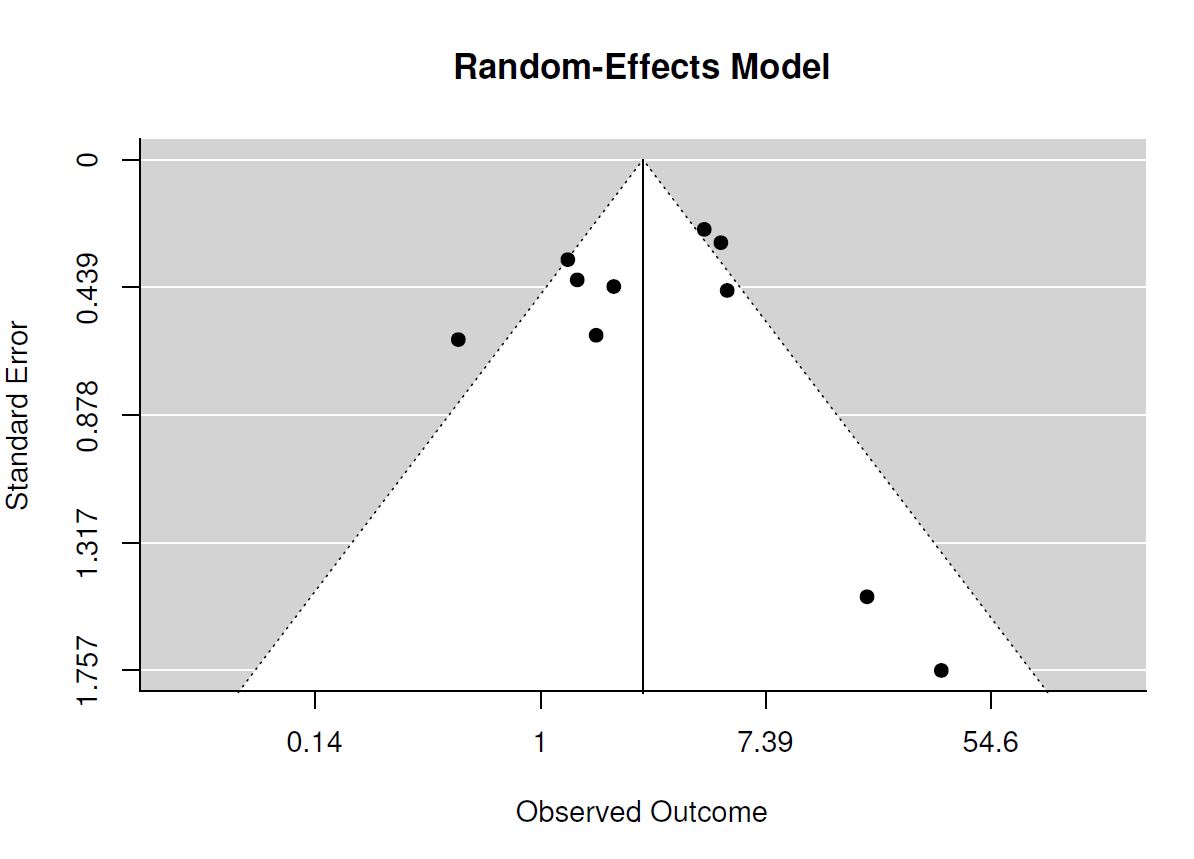

Supplement: Supplementary file 2 — Additional file 2: Figure S2. Funnel plot for cartilage injury. No evidence of asymmetry was observed (p = 0.618). [file 43019_2020_86_MOESM2_ESM.jpg]

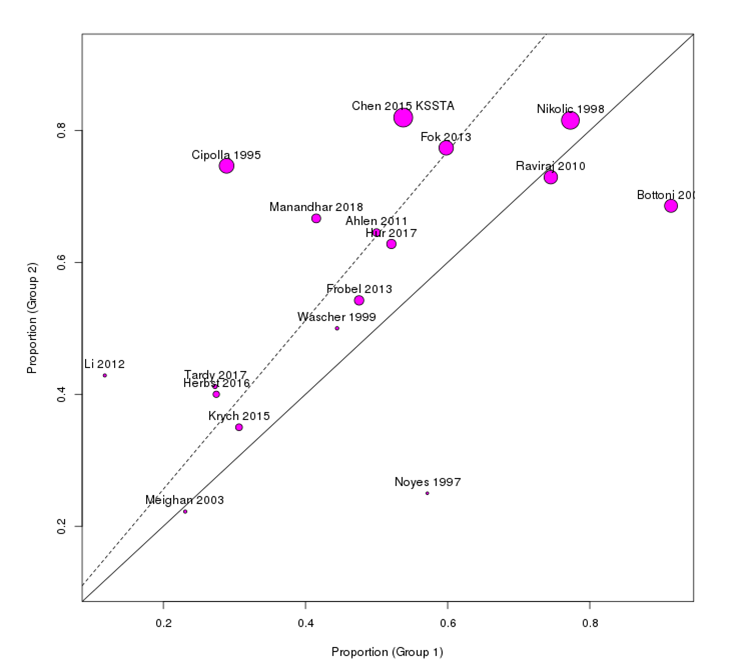

Supplement: Supplementary file 3 — Additional file 3: Figure S3. L’Abbé plot of meniscal tear indicated moderate heterogeneity among the included studies. [file 43019_2020_86_MOESM3_ESM.tif]

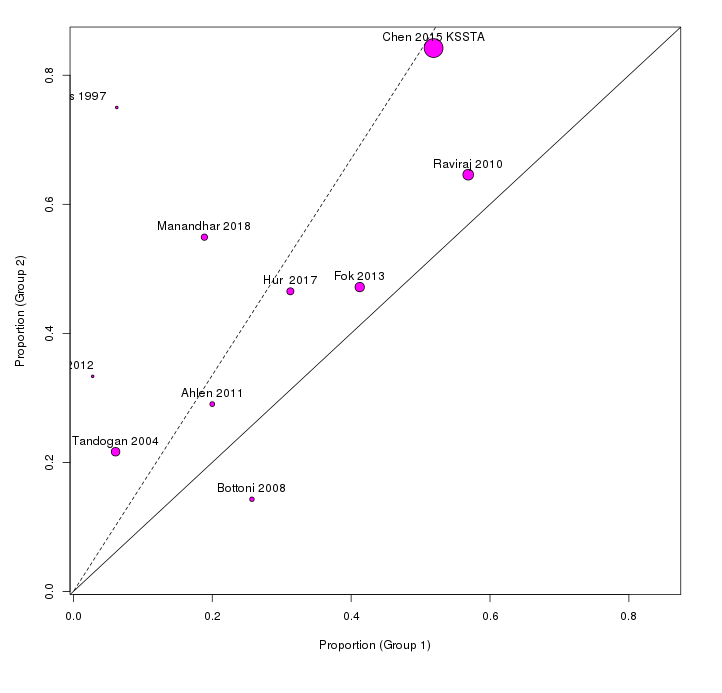

Supplement: Supplementary file 4 — Additional file 4: Figure S4. L’Abbé plot of cartilage injury indicated moderate heterogeneity among the included studies. [file 43019_2020_86_MOESM4_ESM.tif]

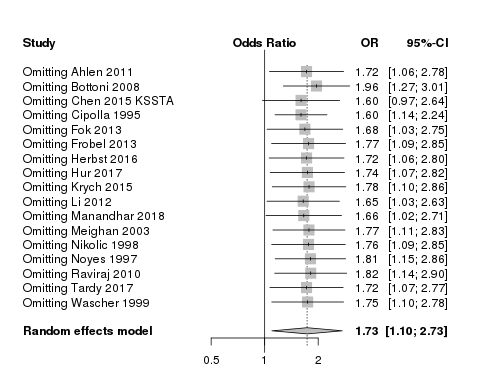

Supplement: Supplementary file 5 — Additional file 5: Figure S5. Forest plot for sensitivity analysis of the risk of meniscus tear. The results were also significant, similar to those in the initial assessment. [file 43019_2020_86_MOESM5_ESM.tif]

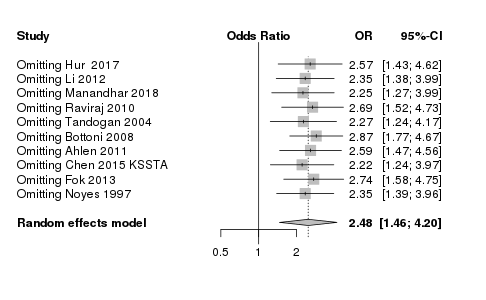

Supplement: Supplementary file 6 — Additional file 6: Figure S6. Forest plot for sensitivity analysis of the risk of cartilage injury. The results were also significant, similar to those in the initial assessment. [file 43019_2020_86_MOESM6_ESM.tif]
